# RESPONSE_ABILITY A Card-Based Engagement Method to Support Researchers’ Ability to Respond to Integrity Issues

**DOI:** 10.1007/s11948-022-00365-6

**Published:** 2022-03-08

**Authors:** Ulrike Felt, Florentine Frantz

**Affiliations:** 1 Department for Science and Technology Studies, University of Vienna, Vienna, Austria; 2 Research Platform Responsible Research and Innovation in Academic Practice, University of Vienna, Vienna, Austria

**Keywords:** Research integrity, Card-based engagement method, Values in research, Borderlands of good scientific practice, Integrity training, Response-ability

## Abstract

Issues related to research integrity receive increasing attention in policy discourse and beyond with most universities having introduced by now courses addressing issues of good scientific practice. While communicating expectations and regulations related to good scientific practice is essential, criticism has been raised that integrity courses do not sufficiently address discipline and career-stage specific dimensions, and often do not open up spaces for in-depth engagement. In this article, we present the card-based engagement method RESPONSE_ABILITY, which aims at supporting researchers in developing their ability to respond to challenges of good scientific practice. The method acknowledges that what counts and what does not count as acceptable practice may not be as clear-cut as imagined and that research environments matter when it comes to integrity issues. Using four sets of cards as stimulus material, participants are invited to reflect individually and collectively about questions of research integrity from different perspectives. This approach is meant to train them to negotiate in which contexts certain practices can still be regarded as acceptable and where possible transgressions might begin. RESPONSE_ABILITY can be seen as fostering the creation of an integrity culture as it invites a more reflexive engagement with ideals and realities of good practice and opens a space to address underlying value conflicts researchers may be confronted with. Concluding the article, we call for caution that addressing issues of integrity meaningfully requires striking a delicate balance between raising researchers’ awareness of individual responsibilities and creating institutional environments that allow them to be response-able.

## Introduction

Over the last decade, the issue of research integrity has received increasing attention. National and international policy documents, extensive calls for immediate action to safeguard the trustworthiness of science, and the creation of special websites, such as *Retraction Watch*,^1^ which report on and offer a forum for debate on recent retractions and the various transgressions against good scientific practice, are all witness to this shift in perception (Biagioli et al., 2019; Hiney, 2015). Research projects addressing integrity issues have been funded both nationally^2^ and internationally^3^ to identify, better understand and develop ways of responding to the challenges of contemporary research. Research institutions, particularly universities, have started to reconsider parts of their research governance, which has often run alongside managerialist logics, creating top-down formalization and increased control over research practices (Mejlgaard et al., 2020).

By now, most universities have not only formulated more explicit integrity guidelines, they have also introduced (obligatory) courses to address issues of good scientific practice (Abdi et al., 2021). However, these trainings are often guided by an ‘awareness-leads-to-avoidance’ logic, which links scientific misconduct to an information deficit on the side of researchers. While communicating expectations and regulations related to good scientific practice is important, criticism has been raised that integrity courses do not sufficiently address discipline and career-stage specific dimensions, and that online teaching with little face to face interaction is not offering sufficient practical experiences. Furthermore courses were criticized as being frequently designed around forms of learning that are too passive to be effective (Phillips et al., 2018; Sefcik et al., 2020). Analysts also underline the importance to embed this addressing of integrity issues into the overall socialization of novice researchers, which would require commitments on many different levels (Hyytinen & Löfström, 2017; Mejlgaard et al., 2020; Orr, 2018; Todd et al., 2017).

Therefore, numerous analysts also call for moving beyond prescriptions and rules to make the concept of research integrity meaningful in researchers’ work (e.g., Sarauw et al., 2019; Young et al., 2018). New approaches to integrity training are being presented^4^ that acknowledge how challenges to good scientific practice need to be seen as entangled with developments in contemporary research environments (Pizzolato et al., 2020). For facilitators of integrity training, this means opening up matters of integrity towards more interactive engagement (e.g., Jagiello-Rusilowski, 2017; Lewis, 2020; Tokalić & Marušić, 2018) and centering their efforts on participants’ concrete experiences and concerns. This could then avoid trainees developing a ‘ticking-off the integrity-course-box mentality’ (Phillips et al., 2018), where they perceive integrity courses more as a burden than as a chance to reflect on their work as researchers with a responsibility to their communities and societies at large. Acknowledging that the development of integrity skills is a complex *process*, which needs time and careful contextualization, is thus seen as an essential element in developing a robust research integrity culture (Barak & Green, 2020; Hiney, 2015; Sarauw et al., 2019).

Studies in recent years stress that transgressions of good scientific practice rarely present themselves as the black and white issues guidelines and rules may suggest them to be (Davies, 2019; Hangel & Schickore, 2017). More frequently, researchers express wider concerns about the discrepancies between ideal understandings of science and their lived experiences in contemporary academia (Buljan et al., 2018; Felt, 2009; Müller, 2021) and have to find ways to navigate what we call the “borderlands of good scientific practice*”* (Felt, 2016). The concept of borderlands refers to the fact that, on the one hand, what counts and what does not count as acceptable practice may not be as clear-cut as imagined. And on the other hand, the concept also indicates that it is researchers who are asked to negotiate (with themselves and others) in which contexts certain practices can still be regarded as acceptable and where possible transgressions might begin. Embellishing of visual representations, for example, has sparked quite some debate about where the limits of acceptable modifications are situated. And so did numerous other practices. Thus, a more nuanced understanding of integrity issues must also coincide with attention to the research environments and cultures researchers find themselves in (Aubert Bonn & Pinxten, 2021; Biagioli, 2019; Haven et al., 2020; Wellcome-Trust, 2020). Contemporary ways to organize and reward research such as the need to play the “indicator game” (Fochler & de Rijcke, 2017), persistent logics of hypercompetition (Fochler et al., 2016), growing temporal pressures (Felt, 2017b), and occupational insecurities (Sigl, 2016) are seen as some of the main factors that potentially ‘support’ transgressions against academic integrity (François et al., 2020; Metcalfe et al., 2020).

Embedded in these debates, this article aims to contribute to the growing pool of reflexive integrity training tools by proposing a card-based engagement method we call “RESPONSE_ABILITY.” The development of the RESPONSE_ABILITY method derives and builds from our research as science and technology studies scholars who study how integrity issues become a matter of concern in researchers’ “epistemic living spaces” (Felt, 2009). The latter concept refers to the importance of the structures, contexts, rationales, actors, and value orders which mold, guide and delimit researchers’ potential actions. Therefore, we understand the ways in which ‘good’ knowledge is produced as inseparably intertwined with how researchers (can) live in science.

The article proceeds as follows. First, after a brief outlining of our understanding of the issue at stake and an embedding of our approach into social science methods using cards as stimulus material, we describe the development and validation protocol of the card-based method. This is then followed by a detailed description of the method’s choreography and the cards utilized during the activity, including our argument for why we propose this particular choreography and choice of cards. We then discuss the moderation and preparation needed to use the RESPONSE_ABILITY method for integrity training. Finally, we provide examples from the discussion dynamic this method facilitates to demonstrate the potential for this form of engagement to sensibilize trainees to the multiplicity and contextuality of transgressions against good scientific practice while underlining the importance of the participants’ engagement with each other. The conclusion will then briefly review the potential of the method and point to some of its limitations.

## RESPONSE_ABILITY: A card-Based Discussion Method

### Contextualizing the Method

Before we describe the RESPONSE_ABILITY method in detail, it is important that we make explicit the conceptual considerations that are inscribed into it (Law, 2004). Integrity is understood by us not solely as a fixed set of rules and norms, but as a lived practice that is part of the “boundary work” (Gieryn, 1983) researchers engage in when doing research. Here, boundaries refer to the field-specific delimitation of what is a relevant question, accepted methodological approaches, publishable entities, or career-relevant achievements. Boundary work, of course, also includes the question of which research practices still count as acceptable, and which do not. As mentioned above, in our research, we develop the concept of borderlands of good scientific practice. With this spatial metaphor, we are acknowledging that researchers encounter fuzzy, situated, and sometimes shifting boundaries for what qualifies as good practice during their epistemic and social journeys through the ever-changing research landscape. As researchers continually push the frontiers of knowledge, they will necessarily encounter uncharted territories, where they must assess what constitutes legitimate scientific practice and what does not (De Vries et al., 2006). In addition, we should also be aware that with changing methodological possibilities, well-known practices can change.

Therefore, the aim of the RESONSE_ABILITY engagement method is to train researchers’ ability to respond to such challenges in their everyday practices and incentivize them to ask specific questions. Thus, we aim to support the cultivation of reflexive skills and sensibilize researchers to both questions of value choices within research and to the structural boundary conditions they find themselves in. By talking about the *ability to respond*, we want to draw attention to the concrete actions researchers take when they are confronted with challenging situations and to acknowledge that a unique set of skills is required to verbalize concerns and act adequately within these situations (Felt, 2017a; Schrader, 2010). Moreover, we conceptualize the idea of *response-ability*, in line with feminist research, as a care practice describing the “willingness to respond” (Martin et al., 2015: 634) to matters of concern without necessarily prescribing what a response should look like. Therefore, by using this engagement format, we invite researchers to individually and collectively reflect and experience what it means to navigate the borderlands of good scientific practice.

### Using Cards as Stimulus Material

The card-based engagement method RESPONSE_ABILITY is grounded in a history of social science research that uses cards as stimulus material. There is, however, no single tradition but rather a broad spectrum of experimental methodologies that use cards to open up, steer and structure discussions, most notably with focus groups and interviews. The functions of cards range from being mere discussion inputs to more elaborate choreographies in which participants sort, choose, position, or even create cards (Rowley et al., 2012). Several of the new interactive integrity training methods use card-based inputs for stimulating discussions. Luger et al., (2015), for example, present a card game that is used to train the reflexive capacities and competencies of designers who deal with issues related to data protection. Tokalić and Marušić (2018) use a card game to facilitate discussions on ethics and integrity in peer review processes. Meanwhile, the “Dilemma Game,” developed at the University of Rotterdam (van Donzel et al. 2013), addresses research integrity by discussing specific dilemmas in research.

While when developing the card-based engagement method RESPONSE_ABILITY we have explored these engagement methods, in terms of concrete choreography and specific methodological considerations it primarily draws from the card-based discussion methods IMAGINE (Felt et al., 2014) and IMAGINE RRI^5^ (Felt et al., 2018). Both methods the former when addressing matters of concern related to nanotechnologies with citizens, the latter for reflecting on the meaning of Responsible Research and Innovation (RRI) with researchers—take specific care to break complex issues down into “micro-moments” (ibid.: 207). Thus, both methods support addressing issues at stake in great depth and use the group setting as a resource to engage with each other.

Drawing from these methods, we decided to develop a specific form of card-based method as observations in previous projects have shown that the presence of cards at the discussion table (Felt et al., 2014) has several advantages. It allows us to move away from classical question-guided discussion groups towards more self-reflexive engagements, which foreground participants’ experiences (Mammen et al., 2016). Each participant can individually choose—out of a broad repertoire of cards—which issues they want to address and which not (Sutton, 2011); thus, they collectively remain in command of the direction the discussion takes. The material presence of information, statements, and short stories in the form of cards raises awareness of the breadth of potential issues and invites the participants to actively position themselves. The cards present a broad spectrum of information in a graspable manner, thereby supporting participants to talk about a topic even though they might not have experienced the described situations themselves (Bandaelli, 2010). Moreover, the cards allow us to address sensitive issues, which might otherwise be regarded as taboo topics and thus, not given adequate attention (Olesen et al., 2018). Finally, because topical input to the discussion comes through the cards, this also gives the moderator more space to focus on facilitating the discussion.

### Developing and Validating the RESPONSE_ABILITY Method

The development and validation process of the RESPONSE_ABILITY method follows a clear protocol. We started with a careful pre-study of the issues at stake that need to be addressed through an extensive literature review on research integrity debates. We also made use of the insights from numerous previous discussion groups and interviews on work and life in contemporary academic research performed by one of the authors (e.g., Felt, 2009; Fochler et al., 2016). This was followed by deciding on the choreography, i.e., on the different steps through which issues at stake would be addressed (these are the stages in the discussion, generally 3–4 in total) and by developing a first set of cards. After testing these first card sets with members of our own research community and students, the card sets were refined and the choreography was adapted. The card-set which we provided as supplementary material for this article went through several of these circles of assessment and refinement until we reached a point of saturation. This occurred as we found the most efficient choreography, number of phases, content, and number of cards needed to open up participant engagement and spark in-depth debate.

To validate the impact of RESPONSE_ABILITY, we asked participants to write short reflection papers after participating in our card-based sessions to better understand how they experienced the engagement and what their takeaways were. This provided us with detailed insights into what the method achieved and where, as with any method in this domain, its limitations lie. It will, however, be key to consider disciplinary particularities and adapt the content of the cards as needed before conducting a discussion group in different contexts.^6^

### Choreography of Engagement

RESPONSE_ABILITY aims to open up a space where (early-stage) researchers can individually and collectively reflect on how issues of integrity come to matter in their research lives. This space takes shape through a specific choreography that brings together a series of individual moments of reflection and selection/ordering, followed by phases in which the participants are encouraged to share their visions and negotiate individual and collective positions in a discussion with each other. To achieve open engagement with integrity issues, the discussion is structured into four phases, each of which has a set of cards that addresses a specific perspective related to research integrity.^7^ The participants are thus invited to look at research integrity from different angles without predefining or restricting the direction that their reflections will take.

**Fig. 1:**
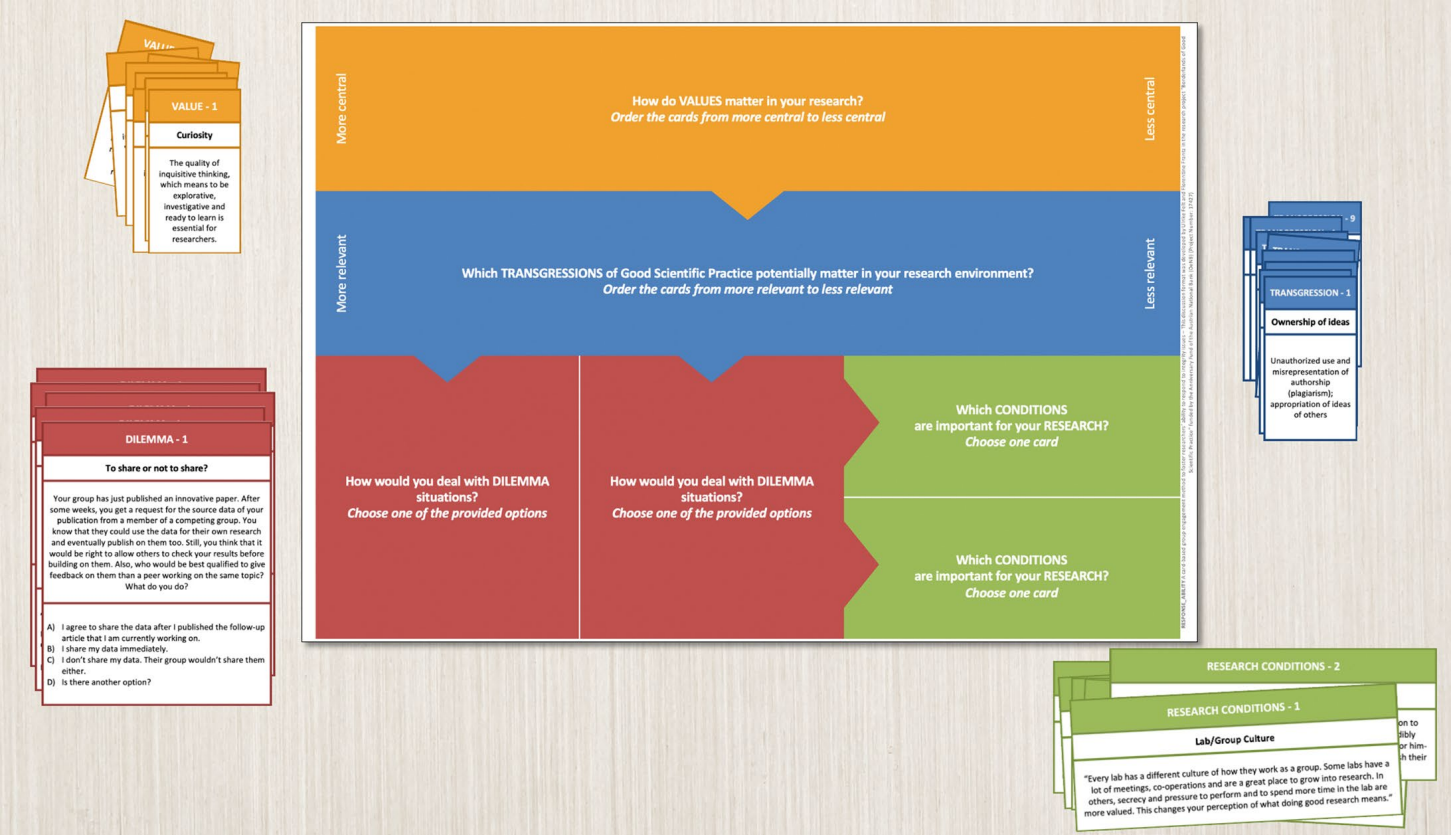
Discussion map and four sets of cards

Each participant has a personal board that indicates the different phases in the form of topical fields where they place the cards that they choose at the beginning of each phase (see Fig. 1). For every set of cards, the participants are first asked to individually rank the cards according to specific criteria or choose a specific number of cards that they are especially keen to discuss. After this moment of individual engagement, the participants are asked to present their choices or argue the outcome of their ordering to the group. This generally triggers a vivid discussion between the participants about their specific choices/orderings, how they relate to different work experiences, and much more. Going through all four phases requires a total of approximately five hours. The alternation between more active discussion phases and moments of introspection in which the participants read and silently choose new cards is a rhythm that allows the participants to truly engage with the questions that come up.

### Types of Cards

RESPONSE_ABILITY uses four sets of cards which we present in the order of their appearance in the discussion. For each set, we describe the kind of work we are inviting the participants to do and point to the reflections that we aim to generate by using them. 
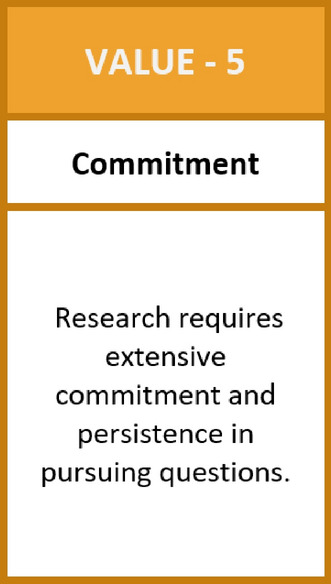


The engagement exercise starts with *value cards*, which cover a broad spectrum of (mostly) positively connotated features, competencies, or attitudes such as fairness, diligence, curiosity, skepticism, commitment, or productivity. Each card contains a short explanation to support participant reflection. We ask the participants to order the cards on a scale from those that are more central to those that are less central to their specific research environment. The participants are thus invited to carefully think about their value ecologies and the tensions between their visions /values, their identities, and what they think is expected from them institutionally within their labs/groups as well as through wider (policy) discourses. They are asked to make these reflections visible by *ranking the cards*. We deliberately decided to start reflections with more positive values to create a framing to which they could relate when discussing transgressions. This approach is in line with more recent initiatives that focus on virtues and values (Marušić et al., 2019) instead of going straight into defining and discussing unwanted behavior. Participants are also free to identify additional values by noting them down on empty cards and including them in their ordering. This flexibility is important to give space to *their* personal values and show that the cards are, of course, only a non-exhaustive starting point (Sutton, 2011). 
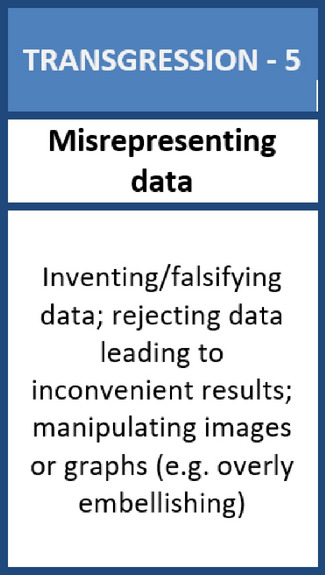


In the second phase, the participants are encouraged to engage with *transgression cards*. These cards point out different and quite widely referenced transgressions against good scientific practices, such as unjustified co-authorship, misrepresenting data, sabotage, and conflicts of interest. These cards represent a spectrum of problematic instances identified in the literature, policy debates, interviews with researchers, and media articles. Again, here we invite the participants to order the cards, this time from what they see as the most to the least relevant in their respective research environments. The term ‘relevant’ was chosen to invite participant reflection about how their specific way of doing research, and their questions and methodologies might allow for different and sometimes new forms of transgressions. During this phase, participants can, for example, think about transgressions that happen most frequently, those that they are most afraid of within their own practices, those that have been at the center of recent controversies in their fields/direct work environment, etc. We will learn about their choice as they argue their orderings. Once again, we offer participants the possibility to add cards for additional transgressions relevant to their work for this phase as well.

While some transgressions seem clearly identifiable, others are more complex to straightforwardly classify as misconduct or acceptable practice. Our approach allows open discussion about how to navigate such borderlands of good practice without moralizing behavior from the start. It also makes room for participants to challenge the cards; for instance, by coming up with situations where a practice that could be regarded as a transgression becomes acceptable. Thus, this approach facilitates interesting exchanges about what counts as good practice in different subfields of a discipline and which of the transgressions participants recognize as being/becoming a (potential) reality for them. 
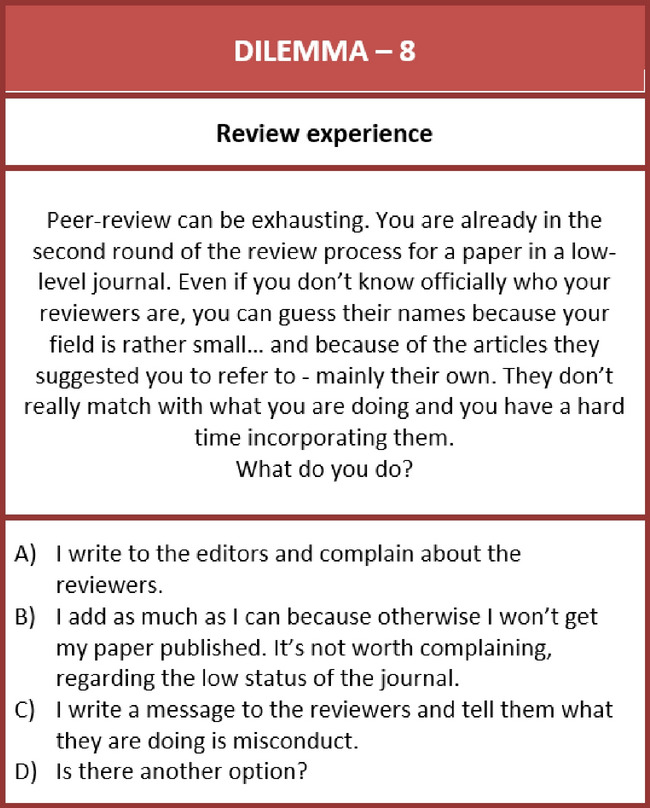


Going beyond ordering work, the third phase addresses concrete dilemma situations. Participants are expected to select one or two of the dilemmas that seem most relevant to them and imagine how they would act during such a situation. These *dilemma cards* contain short stories that describe, for instance, situations about how to react to trouble related to data gathering, errors in a paper already in the publication process, co-authorship disputes, or research biases. The inspiration for using dilemmas as stimulus material comes from the “Dilemma Game” developed by Erasmus University Rotterdam (van Donzel et al., 2013).

Each of our cards briefly outlines a complex situation (3–5 sentences), which points out the tensions that researchers might realistically encounter in research or publication processes. We then list a few potential reactions to support their positioning work. “Is there another option?” explicitly invites responses that go beyond the few mentioned. This phase opens up a reflection on the situatedness and multiplicity of assessments for the given dilemma and is designed to prompt a discussion and exchange about the different real-world experiences that some participants might have had when trying to respond to a dilemma of this kind. 
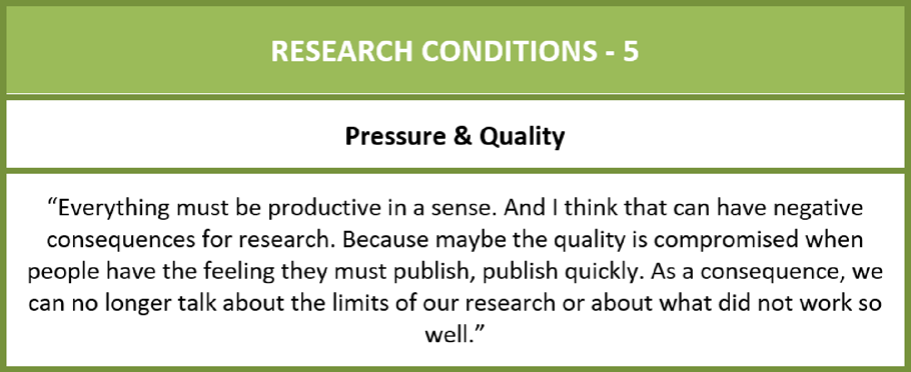


In the fourth and last phase of discussions, the set of cards addresses the *research conditions* seen as key for research integrity. These issues were primarily extracted from the reviewed literature in addition to previous studies on contemporary research conditions by one of the authors (e.g., Felt, 2009). They also took inspiration from the “context cards” used in the IMAGINE RRI discussion groups (Felt et al., 2018). The cards mostly include statements made by researchers about the environments in which research happens and potentially shape how research is/can be practiced. Examples of the issues covered here include the diverse pressures that researchers experience, funding structures, competition, and mentorship. The participants are asked to choose two of the cards they believe are most relevant to them and share how these conditions play out in their research lives and how they might be relevant to research integrity. This phase invites participants to develop a sensibility to how systemic tensions matter within specific research environments when it comes to navigating the borderlands of good scientific practice. While these topics have often already come up during the discussions in previous phases, they mostly did not then trigger in-depth reflection. This will happen in this last phase.

At the end of the research conditions phase, every participant receives blank *change cards* and is asked to make realistic suggestions of how they would change the conditions in which research is happening. The aim of this exercise is not only to invite researchers to deconstruct integrity issues but also to come up with their own constructive ideas. Core to training researchers’ response-ability is the capacity not only to criticize frameworks in which research is happening but also to creatively rethink how (smaller) changes might be implemented to improve the epistemic living spaces they inhabit.

## Moderating the Discussions

The materiality and choreography of the RESPONSE_ABILITY engagement method outlined above structures the discussions into four phases, each focusing on one particular angle of how to reflect on integrity issues. As discussions about research integrity may touch on sensitive topics and open up vulnerabilities, well-prepared moderators are vital for a successful discussion. Below we address specific points touching on the role of the moderator and the preparation for the discussion and its facilitation.^8^

### The Moderator

To assure a safe and open discussion environment, the moderator of the RESPONSE_ABILITY discussion groups should not stand in a direct hierarchical relationship with the participants. Moderators who are supervisors or researchers close to the group may hinder participants from expressing their perspectives freely. Discussion groups should thus, for instance, be led by lecturers of research integrity courses or social scientists/external researchers. Still, however, moderators should have a deeper understanding of the current dynamics of the scientific system and know the institutional environment and debates about research integrity (including regulatory aspects relevant to the addressed field) in order to understand the discussions and guide them in meaningful ways. While moderators should not position themselves as ‘integrity experts,’ they should still be aware of the implicit expert role that the participants will likely ascribe to them. For some moments, moderators will be confronted with questions about how things are to be done ‘right.’ Depending on what the participants are asking, the moderator can choose to either re-ask the question to the group or offer their own perspective. Thus, moderating RESPONSE_ABILITY discussions is a complex balancing act between giving the participants enough freedom to discuss and intensify debates on whichever aspects they deem worth discussing and continuing to guide the participants towards meaningful debates about matters of integrity.

Furthermore, moderating the RESPONSE_ABILITY group discussions requires a certain set of skills: Moderators need to be able to *guide* the discussion while giving enough space to the participants and support their *engagement*. This also includes using subtle positive reinforcements if someone is reluctant to speak up. Moderators should be *careful listeners* to capture not only what people are saying, but also how they are saying it. They are responsible for clearly communicating the choreography and *keeping track of the time*. This includes being attentive to when the participants need breaks and making sure that speaking time is distributed fairly. Moderators should also be able to *deal with unexpected turns* the discussion may take: some cards triggered participants to engage in debates that may not be solely centered on the classical integrity discourses at hand, but still, help to map out the challenges connected to it (e.g., the relation of science and society). It is then the moderators’ task to balance how much room to give to such discussions and decide when to bring the debates back to the initial issues at stake—for example, by starting to discuss the next card. Many of these moderation skills become tacit and embodied when people are experienced teachers and have experience in leading a discussion.

### Preparing RESPONSE_ABILITY Discussions

For organizing the discussions it is vital to consider the group size and composition. The RESPONSE_ABILITY method is designed for smaller groups of ideally eight participants. This number allows enough time for the participants to express their individual views and for participant interaction and the negotiation of positions. In larger groups, there may be too little time for every participant to fully explain, discuss and explore their perspectives, while in groups that are too small—less than five people—it might be challenging to extract a diversity of experiences. It might also be less advisable to compose groups where hierarchies might come to matter. It turned out to be very fruitful to bring together participants who are in similar career stages but are from different groups or even fields. This allows for learning through comparison and fosters deeper reflection while enabling “participatory justice” in the discussion group at the same time (Felt et al., 2018). With participants who have not yet done any research at all, it is challenging to talk about the meaning of research integrity in practice, thus more basic reflective exercises will probably be better suited for them.

Another important aspect for preparing the RESPONSE_ABILITY discussions is the card sets. The card-set that we provided in the supplementary material was developed for interdisciplinary groups and covers a wide range of issues on research integrity. However, when organizing discussion groups, it may be necessary for moderators to adjust the cards to specific disciplinary settings and the research realities of the participants in each group. The cards should always relate and point to real challenges and realistic scenarios that researchers might experience in their day-to-day practices.

Moreover, RESPONSE_ABILITY discussions may offer rich sets of empirical data for qualitative social science research. Analyzing these discussions may yield insights into how participants engage in a situated drawing of distinctions between acceptable and unacceptable practices and towards a more fine-grained understanding of value orders and imaginaries about ‘good’ science at work. Of course, if discussion groups should be recorded, it is necessary to gain permission from the participants beforehand.^9^

### Facilitating the Discussion

To moderate the discussion moderators must familiarize themselves with the card sets and the method’s choreography to be able to explain the overall logic of RESPONSE_ABILITY. At the beginning of every phase, the moderator asks the participants to pick up a pile of cards and perform a specific initial task with them. Once every participant has ordered or chosen the cards (depending on the phase) and placed them on their personal board, the moderator opens the discussion and invites the participants to share their choices and rationales for choosing/ordering their cards. It is essential to give every participant the time and space to talk and to change the order of speaking in the different rounds.

The moderator should encourage the participants to discuss their choices and the reasoning behind them with each other and not enter a dialogue with a subset of participants. This can be achieved by inviting them to share observations regarding the comments of other group members. Here, however, the moderator should not overly intervene, but should rather signal curiosity about the participants’ perspectives, as they themselves are the best to explain how they perceive their research environments and related challenges. Of course, this does not mean that the moderator should not intervene/challenge the participants if they try to justify clearly questionable behavior. In our experience, this has not surfaced as a major problem so far.

Facilitating the discussion also entails thinking about how to keep the discussion going. We suggest that moderators take notes during the discussions with interesting observations that can later be redressed back to the participants. One idea to keep the discussion going in this manner is to address the participants’ card choices and which cards were not chosen—or ranked as less essential. This might make “absent presences” (Law, 2004), i.e., topics people implicitly prefer not to address, visible and steer further interesting discussions. Also, this might trigger exciting reflections in the group as participants compare how others made their choices, for instance, how they clustered cards rather than ranking them (Felt et al., 2014).

## Illustrating Discussion Dynamics

How did participants make sense of the cards and what kinds of interactions did they trigger? We will answer this question by using vignettes clustered along the two main aims of the RESPONSE_ABILITY approach: (1) to train the ability to recognize and engage with situations related to integrity issues, and (2) to make sense of them, situate them in their specific research environments, and develop a position towards them.

The presented vignettes result from an in-depth grounded theory-inspired analysis (Clarke, 2005) of seven discussion groups with a total of 63 participants from the life sciences, physics, psychology, and one interdisciplinary group. The groups were conducted in the framework of our research project in various training/workshop settings for early-stage researchers. While a content-centered analysis of the material will be published elsewhere, the vignettes are used here as tools to portray typical situations through rich description and “vivid accounts of practice” (Jakobsen 2014: 41). Although the names in the stories are fictional, the quotations are taken directly from the transcripts of the discussion groups and were only slightly adapted to create a better reading flow. The participants all gave their permission to be recorded and to be quoted in publications in an anonymized manner.

### Recognizing and Engaging with the Limits of Good Scientific Practice

While analysts have pointed to the fact that mundane research troubles have started to become a growing concern for researchers (e.g., Davies, 2019; Hangel & Schickore, 2017), we also know that it is not always straightforward to recognize when practices cross ‘the line’ and are considered unacceptable. This is nicely illustrated in the following discussion of researchers in biology on (mis)representation of research data.

**Table:** 

“I think this is a problem that is the most common but the least discussed in our field,” Miriam states as she explains why she ranked the transgression card ‘misrepresenting data’ first. She bemoans that there are so many things that researchers do not talk about when it comes to representing data. It is the “little differences” that worry her, the things that are difficult to be standardized and regulated. Her observations trigger a lively debate. Frida continues this line of thought, specifically considering statistical tests, and argues that it sometimes seems arbitrary which statistical test somebody uses. She thinks that people often do not know any better and simply take what works best for them. But she also points to the unlikeliness that her supervisor will critically scrutinize how she performs her analysis: “If you have a polished graph […] and the data fit with what you would expect,” there is usually little discussion about how she did her statistics. Smiling, because Frida apparently describes a situation he recognizes, Alfonso pushes the argument further. A lack of systematic scrutiny is his major concern. He describes himself as trying to be overly cautious with regard to potential tricks that expectations and hypothesis might play on one’s own judgment. One may take “a little step into misrepresenting the data” if you are convinced by a theory. An uncomfortable silence spreads in the room. What to do with this sudden open admission of potential bias? Laura breaks the silence spinning Alfonso’s thoughts further by saying that this is not an individual problem but rather a more fundamental question of how science gets done. In times when scientists need to be good storytellers and create coherent, publishable, mind-blowing stories to survive in academia, it is natural, she argues, that you want to ‘find’ good results. “I mean people most of the time prepare their articles in such a way that they tell a story so that it gets published. And they don’t show the results that could hinder the article [from getting published]."	

This vignette condenses a considerably long discussion regarding the nuances and variants of what potentially constitutes a misrepresentation, and how this is rooted in the different facets of the very environment participants find themselves in. We can observe that for the participants it is not clear-cut where and when ‘the problem’ starts, or how and by whom it should be addressed—how it could and should be handled. Is the source of the problem a lack of discussion or supervision during the research process? Is it a systemic dysfunction in the communication of findings? And, in the end, who should take responsibility for the actions taken? Their exchanges are, however, not limited to concrete practices of presenting results: they become discursively tied to larger structural and organizational features of contemporary research. By taking up different angles on the issues at stake throughout the discussion, participant narratives become increasingly more elaborate and complex as they settle on some converging visions while also keeping certain aspects undecided.

Pressure in science is a recurrent topic in these debates. This is clearly visible in the next vignette where we follow a group of early-stage psychologists and their discussions:

**Table:** 

“Before we discuss the cards you selected in detail,” the moderator says, “I would ask you to guess which [research condition] cards were selected most frequently?” Several people answer, “Pressure and quality.” Some of the participants just point at that very card and nod as it is being raised. Throughout the debate so far, the feeling of being pressured in their work has repeatedly surfaced as have reflections on the threats these perceived pressures may have on the quality of the produced knowledge. Discussing this card, they collectively reflect on “how to get away from this model of publish, publish, publish, and rather focus more on quality”. Elisa shares a brief anecdote of a discussion she followed between two professors. “One professor […] said: “When we were young and doing our PhDs, we had much more time. We didn’t have so much pressure to publish […] For junior researcher it’s so much harder today.” And the other professor who was even younger just said “It will always be like that. The more you do, the better you are, the more you publish the better you are.” She strongly disagrees that doing good research is only about delivering large quantities of knowledge and expresses frustration about the pervasiveness of the capitalist understanding of the world. Paula agrees that her understandings of being a good scientist also do not align with how research is rewarded nowadays. She ties her frustration about the importance of papers to the temporal imaginations that come with them: “You have to do your PhD in three years, you have to publish three studies in three years, and you only get published when you have significant or positive results. I think this is the most hindering thing about doing good research because it really makes you work poorly to get published, but you don’t have the time to really think about the problems and discuss them, because you only have three years.”	

Here, deep frustrations and partial disappointment surfaced, feelings which are often only expressed behind closed doors (Olesen et al., 2018). RESPONSE_ABILITY discussion groups, however, are apparently perceived by participants as a safe space to reflect on how their living conditions relate to integrity issues in more detail. When following these participant stories, it seems that growing into academia comes hand in hand with a de-mystification of science as a practice. In the vignette above, we observed how participants reflected on the alignment of evaluations with contemporary academic market logics, where the sheer accumulation of countable entities is rewarded. Participants concretely identify how this pressure manifests and why they think it may hamper them from doing good work. At other moments in the discussion, they share solutions to handle the emotional moments, they learn through comparing between different experiences, and, thus, they gradually express a much finer-grained vision of what exactly is at stake and how to potentially respond to these challenging situations. This feeling was clearly spelled out as an important insight gained from the participation when giving their feedback to us. Empowering participants to choose the issues they want to focus on and allowing them to trade experiences and potential solutions is thus generally perceived to encourage to take action and develop more proactive positions.

### Positioning Oneself Towards Issues of Integrity

Understanding research integrity as a set of complex matters of concern that are situated in specific research environments means accepting the plurality of reactions necessary to deal with such matters. RESPONSE_ABILITY thus does not hand out readymade solutions to every situation where research integrity is challenged. Rather, we invite participants to position themselves, reflect, speak up and imagine change. We understand positioning work here as an act of participants consciously making their own, often tacit, understandings of good scientific practice and what it means to be a good scientist explicit. These understandings are then expressed, defended, negotiated, and potentially changed through the discussion. Below, we describe three different positioning moments, namely participants’ reflections on which issues should be seen as matters of integrity, how to react to the misbehavior of other researchers, and their ideas about how to re-structure and re-think academia to better support research integrity.

We will start with a vignette from an interdisciplinary group where a participant describes the empty card he would like to add to the debate about values in research. Here, we not only illustrate how the participants negotiate the meaning of the new card but also how they engage in debates about which issues should be considered matters of integrity and which not.

**Table:** 

“Is there anything you missed in our discussion about values in science?” asks the moderator once the participants have each described how they ranked the value cards on their board and the group has already collectively reflected on some of the similarities and differences in ordering rationales. Lorenz proposes a card about “Resilience. […] I think that’s a little bit missing here. I see a lot of facets of the PhD in there, but this is a little bit missing in my opinion.” He goes on to argue that for him it is vital to be able to deal with setbacks and recover from challenges or errors. His suggestion is faced with criticism: “Is that really a value?”, Lucia asks. After all, she does not see it being valued—rather, it is required that individuals come up with a certain mental strength if they want to succeed, survive in science. For Lorenz, mental health and seeing a “person rather than just a scientist” deserves attention in this discussion on research integrity and he keeps on arguing for it. Alfonso agrees and sees the point Lorenz wants to make, but for him, the capacity to continue working despite the problem one encounters is captured by the value of commitment. Lorenz responds that “if you ramp up the commitment to one hundred, problems you have to deal with hit you harder. And if you go down with the commitment, you can deal with problems easier.” Not everyone agrees, but Lorenz continues to argue that it matters to think about the person doing the research and that none of the values on the cards are self-evident but depend on the person behind them. After an intense discussion about what it means that science is conducted by humans, Lorenz closes the debate by stating that his concern would probably have been satisfied by “adding two words on [the] fairness [card]: fair treatment of others *and yourself*.”	

We thus see a concern raised that was not explicitly addressed through the cards: How does researcher well-being matter for research integrity? This vignette illustrates a moment where the participants negotiate which concerns are allowed to belong under the heading of integrity and which not. Thus, they engage in collective boundary and positioning work about where they—as human beings — and their mental health should be discursively situated. Moreover, this short encounter also allows us a glimpse into how the participants negotiate the meaning of the cards and actively challenge them during their positioning work.

A moment of active positioning work can also be observed when participants collectively reflect on how to react to the misbehavior of other researchers, specifically reviewers. In the following vignette, we encounter a group of early-stage physicists.

**Table:** 

“It has happened to me before and it is something that pisses me off very much,” Lena starts by talking about why she chose the dilemma card on reviewer misconduct. Telling someone to cite their own articles as a review comment is a transgression of good scientific practice for her if they clearly don’t match the argument of the paper. While she cautions that sometimes reviewers suggest useful papers which she did not know, she is outspoken about pushing back against reviewer fraud in her response letter to the editor. “I would say that I didn’t find that they were connected enough to the result of the paper to include them.” She perceives the nodding of her colleagues as she looks around. “I also chose the same dilemma,” Kavaan continues shyly, “I experienced this as well and I think it is common practice to agree with everything that the referees ask for, especially their easy suggestions.” He describes his process of first revising their easy comments, such as correcting typos and justifying formulas, before he adds the suggested articles “without even actually reading the whole papers,” Yet he is outspoken about just adding a limited number of papers. Xenia interrupts their discussion. She stresses that she does not know whether or not she would add the references. It would depend on the journal she would be aiming for. Her group recently wanted to publish in a high-impact journal. And they agreed to simply add a sentence to include the reviewers’ paper. “I am pretty sure we wouldn’t have done this, at least I wouldn’t have done this if it was a different journal. […] It is always a compromise.” After being asked by the moderator whether others had also pondered over choosing this dilemma, Gregory takes the opportunity to talk about the problems that his group faced when one of their reviewers was a member of another school of thought. Once he ended describing the complex odyssey of compromising in order to get the paper published, Mia shared an anecdote, partly as advice to Gregory, partly to expand the review problem. “Sometimes acknowledgments are used to avoid certain reviewers, right? So, you just put people into the acknowledgments and then they will not get the paper to review.” Everyone laughs before the discussion returns to more serious reflections on reviewers and power abuse.	

The participants seem to agree that it is wrong for reviewers to demand the inclusion of references that are not useful for revising a paper. Yet, the degrees to which the participants would resist this demand differ considerably and open up a diversity of positions. They collectively ponder about power relations, dependencies, and how these affect their room to maneuver. The spectrum of positioning ranges from response letters with frustrated and emotional complaints to more pragmatic approaches, whereas having little resistance to reviewers’ requests is seen as a way to achieve their own goals. In their discussions, they collectively acknowledge that due to the structures of contemporary academia they may find themselves in uncomfortable situations where it may not be evident which paths to take. Still, they work out a variety of context-specific reactions and exchange their experiences, always with a shared understanding that this should not happen, yet without a clear vision of who could really change these behaviors perceived to be at the border of what can be seen as ethically acceptable.

Finally, we want to briefly zoom in on the last stage of the discussion and show how the participants from an interdisciplinary group express their ideas for how research could/should change to support the conduct of good scientific practice.

**Table:** 

After an extensive round of discussing the research conditions, the participants are asked to come up with changes they want to see in science and write them on the empty change cards. The cards are quickly filled with catchy titles and long lists of changes. Everybody is then asked to present their suggestions. Some of them overlap, such as a general desire to have “more time to really think about what and why are we doing science, how we communicate with others and how we can be open to the ideas of others and the critique of others.” But some participants have more concrete suggestions, such as Anna’s idea to re-think letters of recommendations not only as hierarchically top-down but also as bottom-up: “If it’s about leadership positions, why not [ask for] references from people who have worked below you, who have worked in your team?” She goes on to describe how this could not only help to combat power abuse and make it more visible but also strengthen the value that supervising and leading a team has if it is acknowledged as something that can be accounted for. This idea that the incentive systems are misguiding is also taken up by Eva: “I think most of the problems that we’re dealing here right now could be solved by just having a different incentive structure. So right now, what we do is we reward only things that we can’t control, which is data and results, right? If you have nice results, then you can publish them in Nature and you get a nice position.” She goes on to argue that incentivizing good practices, such as sharing data, also publishing negative data … would, in the long run, benefit science.	

Listening to their whole-hearted pleas and concrete proposals for a better, fairer, and more sustainable science, we can observe that they truly enjoy this exercise. Finishing the discussion on a more positive note is a deliberate decision that should ensure that the participants leave the group without being overwhelmed after hours of talking about challenges and concerns within research. The participants should instead be reflecting on concrete changes they would see for creating viable “response-ability conditions” (Felt, 2017a). Furthermore, talking about research conditions and how even smaller changes can have a great impact is meant to avoid a rigid individualization of responsibility and foster an understanding of the participant’s own agency as embedded within wider systems—thus also giving space to reflect on systemic responsibilities (Davies, 2019). Training early-stage researchers to reflect and eventually respond despite challenging conditions is important not only for training future researchers but for training them as response-able citizens who find themselves in complex worlds, where they will often be asked to act despite complex circumstances.

## Conclusion

In this article, we described the card-based engagement method, RESPONSE_ABILITY, which was developed to facilitate debates on how research integrity comes to matter in the works and lives of early-stage researchers. Guided through four different phases, the participants are invited to take up different perspectives on integrity issues, negotiate what doing good research means for them and how they might deal with transgressions against good scientific practice. Core to the development of this discussion method was the idea to contribute a training tool for the growing body of reflexive teaching materials (e.g., Jagiello-Rusilowski, 2017; Lewis, 2020; Tokalić & Marušić, 2018) that acknowledge the existence of what we call borderlands of good scientific practice. Therefore, RESPONSE_ABILITY is meant to be sensitive to the situatedness of integrity judgements and supporting researchers to develop context-sensitive responses to integrity issues. This capacity to reflect on and react to challenging situations, often under unideal conditions, i.e., to be response-able in situations that pose challenging questions, is what we aim to train with this method.

The feedback we collected after the engagement exercises clearly shows that the participants specifically appreciated the situated, in-depth engagements that this method allows. They underlined the importance of the cards, which opened up issues they had sensed as problematic but did not find ways of addressing/naming them. They are also deeply fond of discovering the subtle differences and similarities that were brought to light by comparing the different experiences of participants. RESPONSE_ABILITY discussions allow participants to learn and experience that often there is not just one good way to solve a problem, but rather several different ones. Participants unanimously stress that they left the discussions with a much finer-grained understanding of the issues at stake, which will potentially allow them to better address the problematic situations they may encounter.

However, no single training tool can completely ensure that early-stage researchers enculture all the skills and knowledge needed to deal with issues of integrity (Sefcik et al., 2020). Methods such as RESPONSE_ABILITY discussions must rather be seen as supporting the continuous process of growing into research in ways where the researcher’s own knowledge production is geared towards creating good knowledge. They work better for early-career researchers who already have some experience in research and are thus already confronted with the complexities of the borderlands of good practice. They are meant to be performed in small groups with well-trained moderators who have a good basic knowledge of the field the participants work in but are not directly related to them—confidentiality is key. Creating safe spaces to individually and collectively reflect on the borderlands of good scientific practice and thus to map out the room for participant maneuvering is thus a valuably reflexive moment. Opening up issues that are often silenced for a nuanced debate in a competitive, accelerated academic world is essential for developing an integrity culture that is robust even under difficult conditions.

Taking early-stage researchers and their concerns seriously as actors who can and should reflect on challenges in research does, however, not mean that responsibility for research integrity is only up to them. Addressing issues of integrity meaningfully means striking a delicate balance between raising researchers’ awareness of individual responsibilities and analyzing the “response-ability conditions” (Felt, 2017a) of the institutional environments they are embedded in. Calls for a change of research frameworks and cultures to support researchers’ integrity efforts are becoming louder in recent years (Valkenburg et al., 2021; Wellcome-Trust, 2020). But the conditions necessary to create thriving research ecosystems—conditions that support researchers’ ability to act in line within the ideals of good scientific practice—are apparently not easily achievable. They instead require constant work.

Following the process of engagements allows us to witness how essential it is to think about where to locate questions of responsibility for research integrity. Indeed, while many measures to improve research integrity are focused on educating the next generation and teaching them the rules, during RESPONSE_ABILITY discussions participants convincingly argue that change needs to happen at both the individual and the structural levels—and in a well-aligned manner. Embracing the undoubtedly uncomfortable idea that transgressions against good scientific practice have always and will always accompany research, it seems imperative to prepare novice researchers to become critically thinking actors who can find a position for themselves in challenging and potentially uncertain situations—capable and ready to adapt their perspectives and actions with the ongoing development of research. Yet, returning to the notion of an integrity culture and building on the experiences gained from these engagement exercises leads us to also call for the creation of an environment in which research practices are addressed, shared, reconsidered, and carefully put in context on a regular basis.

## Footnotes

1 https://retractionwatch.com/.

2 e.g., “Borderlands of Good Scientific Practice,” https://rri.univie.ac.at/en/research/research-projects/completed-research-projects/borderlands-of-good-scientific-practice/.

3 e.g., https://embassy.science; https://eneri.eu/; http://entireconsortium.eu/; http://h2020integrity.eu/; https://path2integrity.eu/; https://printeger.eu/.

4 For a collection of research integrity tools and resources, see: https://embassy.science/wiki/Resources.

5 RRI is the abbreviation for “Responsible Research and Innovation”.

6 Additional thoughts about how to adapt the cards to different disciplinary needs can be found in the supplementary material.

7 The card sets and discussion map are published under a creative commons license, which is accessible under the following link https://phaidra.univie.ac.at/o:1417029.

8 There is extensive supplementary material for this article, which goes into detail about planning and moderating RESPONSE_ABILITY discussions. It also outlines considerations for organizing potential moderator training seminars.

9 Please find more detailed reflections for recording the discussions in the supplementary material.
